# British version of the Iowa test of consonant perception

**DOI:** 10.1121/10.0034738

**Published:** 2024-12-01

**Authors:** Xiaoxuan Guo, Ester Benzaquén, Emma Holmes, Inyong Choi, Bob McMurray, Doris-Eva Bamiou, Joel I. Berger, Timothy D. Griffiths

**Affiliations:** 1 Biosciences Institute, Newcastle University, Newcastle upon Tyne, NE2 4HH, United Kingdom; 2 Department of Speech Hearing and Phonetic Sciences, University College London, London, WC1E 6BT, United Kingdom; 3 Department of Communication Sciences and Disorders, University of Iowa, Iowa City, Iowa 52242, USA; 4 Department of Psychological and Brain Sciences, University of Iowa, Iowa City, Iowa 52242, USA; 5 University College London Ear Institute, University College London, London, WC1E 6BT, United Kingdom; 6 Human Brain Research Laboratory, Department of Neurosurgery, University of Iowa Hospitals and Clinics, Iowa City, Iowa 52242, USA

## Abstract

The Iowa Test of Consonant Perception is a single-word closed-set speech-in-noise test with well-balanced phonetic features. The current study aimed to establish a U.K. version of the test (ITCP-B) based on the Southern Standard British English. We conducted a validity test in two sessions with 46 participants. The ITCP-B demonstrated excellent test-retest reliability, cross-talker validity, and good convergent validity. These findings suggest that ITCP-B is a reliable measure of speech-in-noise perception. The test can be used to facilitate comparative or combined studies in the U.S. and U.K. All materials (application and scripts) to run the ITCP-B/ITCP are freely available online.

## 1. Introduction

Listening to speech in a noisy environment [Speech in Noise (SiN)] is common in everyday life, but it can be challenging for people with hearing impairments. Traditional audiometric assessments of peripheral auditory ability, such as the pure-tone audiogram (PTA), predict SiN,^1^ but they do not capture a large proportion of variance.^2^ Therefore, measures that better simulate real-life listening conditions have been developed to assess sentence-in-noise perception.^3^ SiN measures are an important part of audiometric battery in clinics used to diagnose hearing loss, determine amplification choices, verify hearing aid performance.^4^ They are also widely used in research to investigate cochlear synaptopathy, sound segregation, selective attention, ageing and cognitive impairments as well as other auditory cognitive mechanisms.^5,6^

A potential problem with the implementation of speech-based hearing assessments is that the speech materials might not be suitable for the target population. In the U.K., Parmar *et al.*^12^ reported that only 20.4% of NHS audiology practices give speech tests. This is due to limited clinical resources and the lack of widespread availability of materials geared towards British English. Many commonly used speech tests used in the U.K. are not available in British English. A recent survey of British Audiologists and ENT surgeons^13^ reported that the most commonly used SiN tests for adults were QuickSIN and LiSN-S, and for children was LiSN-S, neither available in British English. We know that listening to accents can disproportionately impact people’s speech processing,^7^ including foreign accents and regional dialects by native speakers;^8^ children are especially influenced.^9^ Aside from speech recognition accuracy, other aspects of speech processing are affected by accent. People’s relative skill at processing SiN does not seem to correlate with their skill at processing accents.^10^ Speech processing takes more effort with accented speech,^7^ suggesting it may tap a distinct set of cognitive and perceptual mechanisms than processing non-accented speech. Consequently speech-based tests across different regions can easily misidentify hearing problems by using a uniform standard.^11^ This highlights the importance of having speech tests in the appropriate accent for clinical applications. From the patient’s perspective, developing a well-designed SiN test is in line with patient identified research priorities in the U.K.^14^

From a research perspective, matched versions of the same tests could bring unique research opportunities. Larger public health-oriented work across U.K.-U.S. can be made possible as well, taking advantage of “natural experiments” to assess the efficacy of various remediation approaches. For example, different criteria for cochlear implantation are used in the U.S. and U.K. A comparison of outcomes of hearing loss across the two populations could help reveal the best implantation strategies.

What type of SiN test would be a valuable addition to the current array of tests? The current zeitgeist in the field is to move towards the most ecologically rich form of such tasks—widely argued to be an open-set sentence-in-noise task. In this type of task, listeners hear a sentence and repeat it back to the experimenter. Such materials are widely available across multiple accents (WILDCAT Corpus^15^) However, some of these skills are not auditory or even perceptual—the seemingly simple task of repeating a sentence is a complex *cognitive* skill requiring lexical access, word recognition, sentence processing, language production along with embedded skills like working memory. The diagnosis of listening problems may be influenced by these skills as they may decline with age or disrupted by developmental disorders even in normal hearing individuals.^16,17^ Therefore, single word tasks may serve a valuable role in controlling some of this non-perceptual variability and contributing to the research and clinical resources. The Iowa Test of Consonant Perception (ITCP) was recently developed to overcome these concerns.^18^ It is a single-word, closed-set task that has a good balance of phonetic contrasts (expressed in the response options for each word) which covers the entire phonetic range of the English language. Each of the options was balanced to equate difficulty and sample a single phonetic feature (place, manner, or voicing). The original test showed very good test-retest reliability, as well as validity based on comparisons with the CNC word recognition test^19^ and AzBio sentence recognition test.^20^

This study sought to develop a British version of the same test using British English speakers with mainstream Standard Southern British accent. This is the modern equivalent of “received pronunciation,” which is widely used in education and the media. The development of ITCP-B aimed at benefiting both clinical practice and research. The ITCP-B leverages the careful work of Geller *et al.*^18^ in identifying an optimal and representative set of items and their response options, and replaces the audio with appropriate British accented versions of each stimulus. We evaluated performance accuracy, the test-retest reliability and the cross-talker validity to assess the reliability of the test itself under laboratory conditions. We also assessed the correlation between the pure-tone audiogram (PTA) and ITCP-B, and the correlation between ITCP-B and a sentence-in-babble (SiB) measure for the convergent validity.^21^ The ITCP-B is free and openly available to the community in the form of a testing APP and scripts that can be easily modified.

## 2. Methods

### 2.1 Participants

Forty-six English native speakers born and educated in the U.K. were recruited for the experiment (30 females, 16 males). Participants were excluded if they had a history of auditory disorders, speech or language disorders, developmental or neurological disorders or were taking psychotropic drugs. Participants were included if they were over 18 years old, and no upper limit was imposed. This is to obtain a representative sample. The PTA averaged across 0.25–8 kHz (in the left and right ears) of the sample was 13.92 dB HL, and the standard deviation (SD) was 8.42 dB HL. The average age was 48.65 (SD = 12.18). Out of the 46 participants, more demographic information was collected on 33 participants on their employment status and levels of education. Approximately 39% of participants had a full time employment, 12% had part-time employment, 33% were retired, 6% were still at university, 3% were a full time parent, and 6% were unemployed. In terms of education, 36% had a postgraduate degree, 45% with an undergraduate degree, 9% with A levels, 9% with GCSEs.

### 2.2 Materials and design

Recordings were made by two native English speakers (one male and one female) with mainstream Standard Southern British accent. There are many accents in the U.K. and the received pronunciation was chosen because it is experienced by the majority of the U.K. population that is exposed to radio and television, even if it is not characteristic of their region.

The word list of the original ITCP test was recorded for each speaker (120 word sets per speaker). These are consonant-vowel-consonant words such as “ball-fall-shawl-wall.” Recordings were made in a sound-proof booth using a large-diaphragm condenser microphone (Rode NT1-A) with a pop filter placed in front. These recordings were made in Audacity (version 3.1.3), with a sampling rate of 44.1 kHz and 16-bit resolution. For both talkers, words were spoken as clearly as possible, at least twice with the carrier “he said [word]” and twice without. This phrase was included to help ensure uniform prosody and rate. Offline, all words were imported into Audacity, the “Clip Fix” function was applied with 95% threshold for clipping and amplitude reduction overall by 5 dB (to allow for restored peaks). Noise reduction was then applied to the entire recording based on the noise profile for a silent period (with 12 dB reduction, sensitivity set to 6.00 and frequency smoothing set to 3). Each word exemplar was marked for cropping at the zero crossing, exported as a .wav file and then scaled to the same RMS level in praat [version 6.2.14 (Ref. 22)] before being re-exported as a final “cleaned” .wav file. The mean duration of the words used was 0.51 s (±0.086 s).

The noise was extracted from an eight-talker babble soundtrack with four male and four female voices that lasts for 15 s in total. Importantly, this babble contained British voices. Segments of the babble noise were taken randomly as a masker for the target word, which were always played 1 s before the target sound and stopped at the offset of the target words. The babble noise was mixed with the target sound with a −2 dB signal-to-noise ratio (SNR).

### 2.3 Procedure

The validation testing of ITCP-B was based on two sessions (sessions A and B). The order of the two sessions was random, subject to participant availability. The two sessions were typically separated by 10 week (median duration = 80 days, range = 5–356 days). In both sessions, researchers carried out separate all three tests in the following order for all participants across session: audiometry, ITCP-B, and SiB tests. The two sessions were identical except for the SiB test: Session A tested the longer version of the SiB test and session B had the same SiB test but shortened by half (this turned out to be unreliable and was not used in this study). Auditory stimuli were presented using headphones (Sennheiser HD 380 Pro) connected to an external sound card (RME FireFace UC). All computer tasks were programmed in MATLAB (R2021a, Mathworks, Natick, MA, United States).

The ITCP-B task consisted of 120 trials in total (shortened by half compared to the original ITCP task), with three blocks separated by short self-paced breaks. The whole test typically took 15 min to finish. Each trial was up to 2 s long with a 1 s inter-trial interval. Half of the target words were spoken by the female speaker, while the other half was spoken by the male speaker. The order of the words was randomised between participants, but the same words were always spoken by the same speakers. The outcome measure used here for the ITCP-B was the proportion of words correctly identified.

The sentence-in-babble (SiB) test was similar to that used by Holmes and Griffiths.^21^ Target sentences were taken from the English version of the Oldenburg sentences and were recorded by a male speaker with Southern British English. Target sentences were structured as name-verb-number-adjective-noun; an example is “Alan brought four small desks.” The background noise was a 16-talker babble that had an onset 500 ms before the target sentence. Participants were asked to repeat all five words from the target sentences: they were presented with a 5 × 10 matrix on the screen and were asked to select each of the five words from a list of ten options. The test used a one-down one up adaptive procedure, with starting SNR at 0 dB and a step size at 2 dB for the first three reversals and 0.5 dB afterwards. The testing consisted of two interleaved runs, where each run had a different set of target sentences and terminated after ten reversals. The median SNR of the last six reversals was taken for each run and both were averaged to compute participants thresholds.

### 2.4 Data Analysis

Data analysis was conducted in SPSS Statistics 29.0.1.0 and matlab R2021a. The results for both sessions were normally distributed, justifying the use of parametric tests. As the overall test design has been established with the previous validation study,^18^ the current study focused on test-retest reliability.

First, as the two sessions were not perfectly counterbalanced, we checked if there were learning effects or other outside influences that could lead to different performance on the two sessions. We compared the accuracy for each test between the two sessions with paired-sample t-tests.

Test-retest reliability was measured the same way as the ITCP validation,^18^ with the intraclass correlation coefficient (ICC), using a two-way random effects model (absolute agreement). ICC is commonly used to estimate the association between variable similar to Pearson correlation, but it considers both correlation and bias when assessing reproducibility.^23^ The absolute agreement measures are used to determine the level of agreement of raters, in this instance the scores of two ITCP-B testing.^24^

The relationship between ITCP-B and other speech and hearing measures was measured using Pearson correlations. Two pairs of correlations were assessed: PTA and ITCP-B (two sessions); ITCP-B and SiB (convergent validity check, for session A only as the shorter SiB was not as reliable). A further cross-talker validity test was conducted by comparing the response to either the male or female speakers. A paired-sample t-test was used to assess if people responded differently to the two voices; the ICC further tests if the test can elicit reliable performance across talkers.

## 3. Results

The mean performance accuracy and standard deviations were extremely similar between the two sessions of ITCP-B: Mean (session A) = 0.68 (SD = 0.08), mean (session B) = 0.67 (SD = 0.09). There was no significant difference in the mean performance between sessions: M_diff_ = 0.005 (SD = 0.043), t(45) = 0.866, *p* = 0. 391. The mean SNR for SiB was −1.07 (SD = 1.44) for session A.

**Fig. 1.**
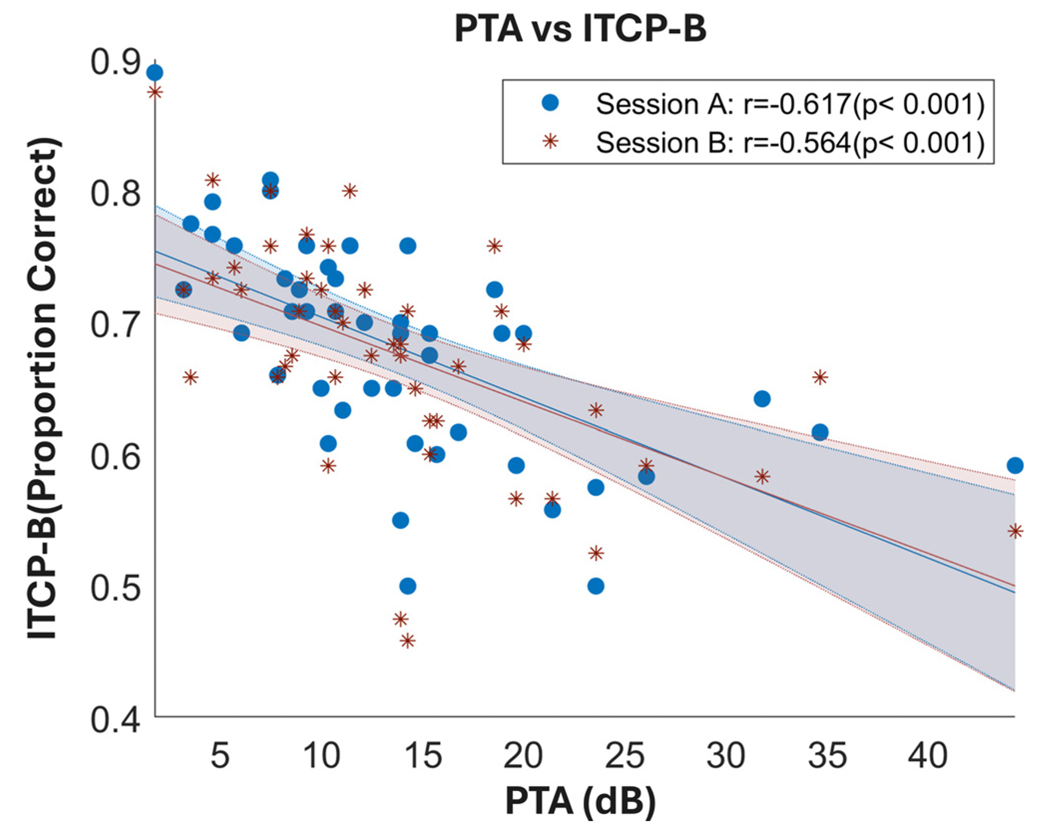
Scatterplot of PTA and ITCP-B of the two sessions. The correlation for session A is in blue and for session B is in red (the lines of best fit and error areas of the two sessions are in their respective colour as well). PTA results were taken from session A. The *x* axis plotted the PTA results in dB SPL, and the *y* axis plotted ITCP-B results measured in the proportion of correct answers overall.

Figure 1 shows the correlation between PTA (averaged across 0.25 to 8 kHz) and ITCP-B. Both sessions had large and significant negative correlations with a similar effect size: r (session A) = –0.62 (p < 0.001), r (session B) = –0.56 (p < 0.001). Note that the negative correlation is predicted since PTA is scaled such that a lower PTA indicates better hearing, while the ITCP-B is scaled such that higher scores indicates better performance.

### 3.1 Test-retest reliability

**Fig. 2.**
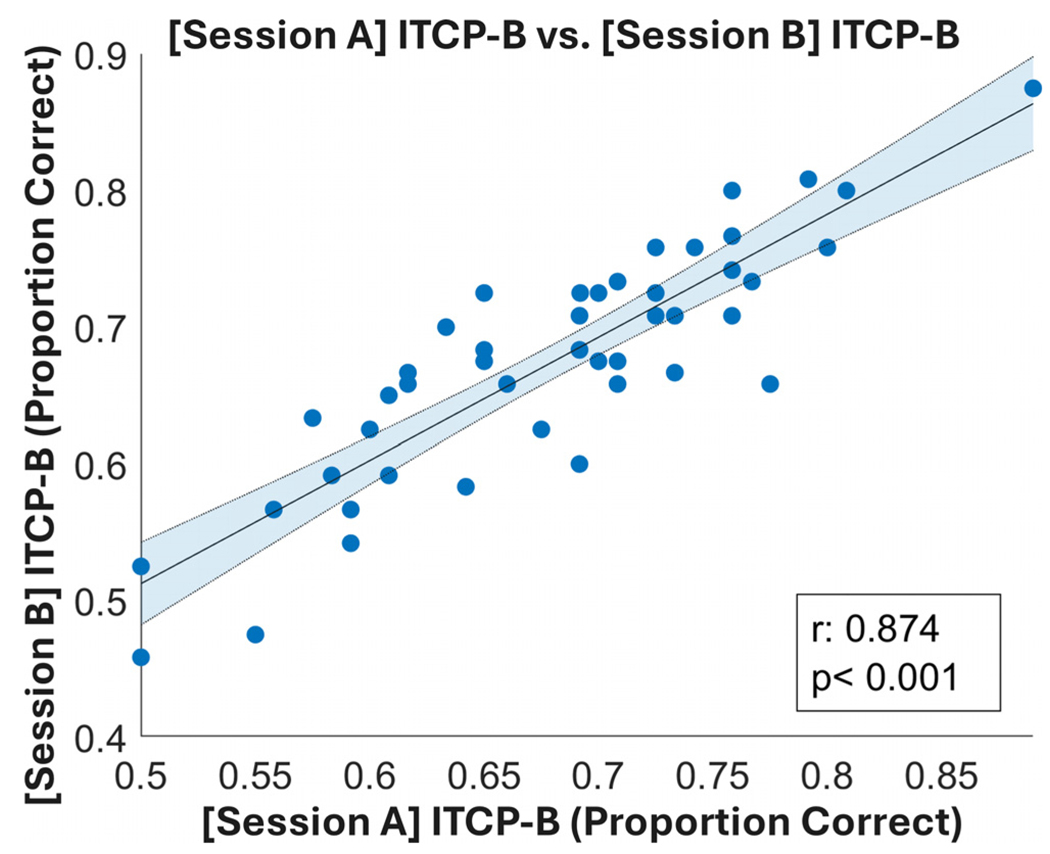
The scatterplot shows the association of the performance on ITCP-B in the two sessions. The *x* axis represents the scores obtained from session A and the *y* axis represents the scores from session B. Pearson’s *r* and a *p* value for a bivariate correlation are shown on the plot as well. The line of best fit is plotted in black with the error area shaded in blue.

We next examined the test-retest reliability of ITCP-B by calculating the ICC between the two sessions. The scatterplot (Fig. 2) displays the close relationship between performance on the two sessions. This is further evidenced by the ICC results (Table 1) that showed excellent reliability of R_ICC_ = 0.93, which exceeds that of the original ITCP test-retest reliability of R_ICC_ = 0.80.

### 3.2 Cross-talker validity

The cross-talker validity test showed that responses on the two sessions to either the female or the male voice did not differ significantly [M (female talker) = 0.68, SD (female talker) = 0.07; M (male talker) = 0.67, SD (male talker) = 0.08; t (45) = 1.82, p = 0.075]. ICC showed a good reliability score as well: R_ICC_ = 0.79, p < 0.001.

### 3.3 Convergent validity

**Fig. 3.**
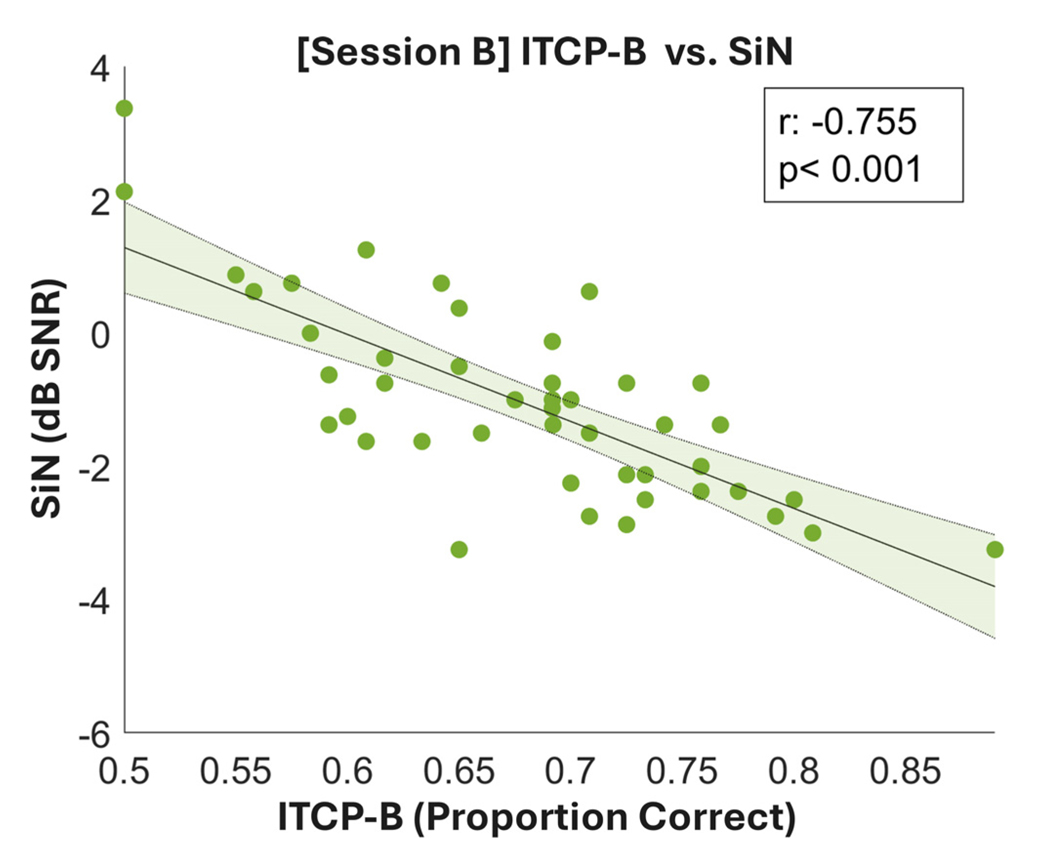
Scatterplot for bivariate correlations between ITCP-B and SiB. ITCP-B results are in proportion correct (*x* axis) and SiB in dB SNR (*y* axis). The line of best fit is plotted in black with the error area shaded in green.

The correlation between ITCP-B and SiB was −0.76 (*p* < 0.001), see Fig. 3 for details. As with the PTA, SiB is scaled as a threshold, so the negative correlation is predicted.

## 4. Discussion

The performance data of ITCP-B showed a Gaussian distribution and achieved a reasonable level of accuracy (around 68% compared to 73% correct reported in Ref. 18). Thus, the ITCP-B meets the minimal criteria for useful measure. While one of the goals of this study is to establish a test that can elicit comparable results from the U.K. and U.S., the performance accuracy of the current study cannot be directly compared with the ITCP results as the subject cohort and test parameters used here were not tightly matched with the ITCP study (which was validated online and tested all words in four subjects). To develop an equivalent test across the U.K.-U.S., further studies are needed which better align the detailed design of the study and the subject populations.

Further, the comparison of the mean accuracy between the two sessions showed no significant difference on performance of the two sessions. This means it is unlikely that the results were contaminated by learning or order effects. This is in part due to the unique design features of ITCP in which each of the four items that comprise a response set are used as the target (and they can be used multiple times across talkers). Consequently, subjects cannot learn which item is the correct response for a given set—they must process the stimulus.

We also demonstrated that PTA could predict ITCP-B performance in both testing sessions, which is consistent with our hypothesis and the literature discussed previously.^1,25^

Both the bivariate correlation and the ICC outcome demonstrated excellent test-retest reliability (ICC = 0.93). This means that the ITCP-B test can obtain a representative and stable assessment of SiN ability over time, which allows for both cross-sectional or longitudinal studies. Again, the ICC score is consistent with the previous results from Iowa (ICC = 0.80), but higher test-retest reliability was obtained in this study. One potential explanation of the higher ICC score in this study is that the validation for ITCP-B took place in laboratory conditions, but the ITCP validation test was carried out online where audio presentation, background noise and distraction cannot be as well controlled. A comparison of online and lab testing carried out by Bridges and colleagues found that online testing for both visual and auditory modality tended to generate lower precision and more variability in performance.^26^ The researchers argued that such discrepancy in results between the two modalities would not invalidate online auditory research, but it did mean that validating online results was necessary. As the current analysis relies heavily on performance stability, it is expected that a more controlled environment will lead to higher ICC score. However, the online ITCP still achieved a very good ICC score (0.80), suggesting that the test can be reliably used online as well as in the lab.

The cross-talker validity assessments were carried out to ensure that each talker was representative of the whole. This was important as to obtain a shorter test, half of the stimuli were presented in each voice this contrasts with the original ITCP where full list of words was heard in both female voices). The shortened version is good for time-limited testing in the clinics but raised a concern over potentially less balanced results. However, the non-significant t-test showed that the shortened version can provide a reliable assessment of people’s SiN ability. Another possibility is that the reduced trial set size in the current study may have been beneficial due to less within-task fatigue.

A further assessment of the validity of ITCP-B against the SiN measure found that ITCP-B correlated strongly with the Oldenburg sentence-in-noise measure. The negative relationship suggested that lower SiN thresholds (better SiN performance) correlated with higher percentage of correct performance on the ITCP-B task. The strong correlation here suggested that ITCP-B can provide an assessment that is consistent with a well-established sentence measure. This finding is consistent with the ITCP study,^18^ which has established a strong association between ITCP and other standardised SiN tests based on sentences. This consistency in the correlation of ITCP-(B) with other SiN measures in the two validation studies (U.S. and U.K.) suggest that first, the results are less likely to be due to other non-specific effects such as motivation and arousal. Second, the ITCP-B can give very similar clinical assessment results to patients’ real-world listening ability despite that sentence-level SiN measures are thought to be more ecological. The fact that this closed-set word-level SiN test is shorter and engages a “purer” auditory speech segregation process also adds to the benefit of using the test when sentence-level tests pose a problem.

As highlighted earlier, the development of a comparable speech-in-noise test in the U.K. and USA would allow for comparisons between two countries with very different criteria for interventions. The ITCP-B and the ITCP potentially represent two tests that can serve this purpose. However, the current experiment only assessed the reliability of the test. To establish age-scaled normative scores, further testing is needed on a wider population, including a wider range of age and hearing sensitivity.

A limitation to the study is that the sample size used is relatively small. Despite having a strong prior, the current sample size is only half of what was used in the original ITCP study.^18^ Further validation study is needed with a larger sample of normal-hearing adults of all age range to establish normative scores for different age groups.

In conclusion, this study shows that ITCP-B test has excellent reliability, convergent validity, and cross-talker validity. The shortened version as used in this study provides a good solution for a quick clinical SiN assessment. The full version can be used for research across the U.K. and U.S. for a more comprehensive test. Both versions are freely available on our OSF page, and researchers can tailor the test based on their preferences.

## Figures and Tables

**Table 1.** Summarises the ICC results from this study (ITCP-B) and the previous validation study [ITCP (Ref. 19)] for comparison. ICC is the intraclass correlation coefficient. CI is the confidence interval, and P is the significance level.

	ICC	CI lower	CI upper	P
ITCP-B	0.93	0.88	0.96	P < 0.001
ITCP	0.80	0.70	0.86	P < 0.001

## Data Availability

The data that support the findings of this study are available upon request. The desktop application for running the ITCP-(B) test is freely available at https://osf.io/tqjuk/ (E.B., X.G., T.D.G.). The MATLAB scripts for constructing your own versions of ITCP-(B) are available at https://osf.io/53jsg/ (X.G., E.B., E.H., I.C., J.I.B., T.D.G.).
